# Determinants of knowledge, attitude and practice in patients with both type 2 diabetes and chronic kidney disease in Fiji

**DOI:** 10.12688/f1000research.18188.3

**Published:** 2019-05-01

**Authors:** Mohammed Alvis Zibran, Masoud Mohammadnezhad

**Affiliations:** 1 School of Public Health and Primary Care, Fiji National University, Suva, Fiji

**Keywords:** Knowledge, Attitude, Practice, Type 2 Diabetes Mellitus, Chronic Kidney Disease, Determinants, Fiji.

## Abstract

**Background: **In Fiji, Type 2 diabetes mellitus (T2DM) and Chronic kidney disease (CKD) are amongst the top four causes of premature mortality, disability and death. This study aims to identify the determinants of knowledge, attitude and practice (KAP) in T2DM patients with CKD in Fiji in 2018.

**Methods: **A cross-sectional study was conducted at Sigatoka Sub-divisional Hospital (SSH) in Fiji in July-August, 2018 using a self-structured questionnaire to test KAP of 225 patients. The inclusion criteria were confirmed T2DM patients (Fijian citizens) with CKD, aged 30 years or above and attending Special Out-Patient's Department (SOPD) at SSH. Independent t-test and ANOVA was used to test differences between demographic variable and practice score while non-parametric tests were used for knowledge and attitude. Spearman correlation and multiple linear regressions were conducted. All tests were set at 5% level of significance.

**Results: **From 249 questionnaires distributed, 225 responded thus response rate was 95%. The mean KAP level was high: knowledge, 23.3/30 (SD±3.25); attitude, 23.1/30 (SD±2.73) and practice, 7.1/10 (SD±2.04). A high level of knowledge was seen in those with university-level education (p<0.001), unemployed (p=0.05) and high average monthly income (p=0.03). Those aged 61-70 years had a 0.53-point lower attitude score (p=0.05) than other age categories, while those >70 years had a 1.78-point lower attitude score (p=0.01) than other age categories. Fijians of Indian descent (FID) had lower attitude (p=0.002) and higher practice (p=0.001) scores.

**Conclusion: **Patients with both T2DM and CKD at SSH have high levels of KAP. The determinants of KAP have been shown and thus, this study identified high-risk groups for low KAP, which can become the focus of future public health intervention.

## Introduction

Type 2 diabetes mellitus (T2DM) is characterized by fasting blood glucose of more than 7 mmol/L or random blood sugar of more than 11 mmol/L in the presence of symptoms of increase thirst/hunger, frequent urination and weight loss ( O’Neil *et al*., 2012). T2DM is the seventh-leading cause of death globally ( Zheng *et al*., 2018), and various complications arise as a consequence of this disease—one of the major ones being chronic kidney disease (CKD) ( Idris *et al*., 2018; Yakush Williams, 2017). CKD is defined as estimated glomerular filtration rate (eGFR) of less than 60 ml/min for at least 3 months ( Bouchard *et al*., 2010) and it is the ninth-leading cause of deaths globally ( Rifkin *et al*., 2012). Fiji’s STEP-wise Surveillance Report (STEPS) of 2011 showed that 15.6% of the adult (25–64 years) population had raised fasting blood sugar ( Snowdon & Tukana, 2011). Since T2DM is the major cause of CKD, accounting for 44% of all cases and hence a rise in T2DM will lead to a greater burden of CKD in Fiji ( Atkins & Zimmet, 2010). T2DM and CKD were found to be among the top four causes of premature deaths and death and disability combined in Fiji ( IHME, 2016). The final stage of CKD is very costly since it requires renal dialysis optimally three times a week and renal transplant eventually whereby cost of each dialysis session in Fiji can range from $USD70 to USD120 ( Consumer Council of Fiji, 2017). Apart from these costs, the healthcare expenses related to management of these medical conditions are borne by the government of Fiji and hence reducing these medical expenses for Fiji’s health ministry as a whole via addressing these 2 diseases from a public health perspective would be beneficial ( MoHMS, 2015).

The knowledge, attitude and practice (KAP) survey, which uses scales and items to gather information from the patients on specific aspects of certain conditions ( Kaliyaperumal, 2004), is a useful tool for assessing and improving control of patient’s disease, delaying associated complications ( Ghannadi *et al*., 2016), influencing better health policy ( Stanifer *et al*., 2015) and increasing awareness for disease prevention. Various factors have been linked to level of KAP in T2DM patients with CKD; for instance female gender has been linked to poor knowledge but good attitude ( Yusoff *et al*., 2016) while males have been shown to have good practice ( Stanifer *et al*., 2016). Similarly, employed participants were found to have higher knowledge while married respondents reported high attitude and practice ( Mutiso *et al*., 2011; Thirsk *et al*., 2014; Yusoff *et al*., 2016). Consequently, a KAP survey of individuals with T2DM and CKD will provide an insight on the current status of the level of KAP and its determinants in a referral hospital in Fiji- Sigatoka Sub-divisional Hospital (SSH), which can be utilized to inform public health programs and help target high-risk cases to improve awareness, promote self-control in patients and reduce or delay complications from T2DM and CKD ( Ghannadi *et al*., 2016).

This is the first study on KAP of T2DM patients with CKD in Fiji which aimed to identify the determinants of KAP in T2DM patients with CKD in Fiji in 2018. The specific objectives were to identify the level of each of the aspects of KAP and to investigate for significant links between the level of KAP and the socio-demographical features in T2DM patients with CKD in Fiji in 2018.

## Methods

### Design and sample

This research applied a cross-sectional, quantitative design to identify determinants of KAP in patients with T2DM and CKD at SSH from 1st July 2018 to 31st August 2018. The inclusion criteria for the study sample were patients with confirmed T2DM plus CKD, attending in the waiting area in front of the Special Out-Patient’s Department (SOPD) clinic at SSH, citizen of Fiji, age ≥30 years and they had to agree to participate in the study. The exclusion criteria were patients with CKD but not T2DM, patients who were incapable of completing the questionnaire, and those who were not interested in taking part in the study.

The study was conducted at SSH’s SOPD clinic. SSH is a Sub-Divisional Hospital in the Western Division of Fiji and it is a secondary-level hospital which provides general outpatient services, SOPD, inpatient services, maternity, child-health, eye-care, laboratory tests, radiological examinations and pharmacy. It is the only hospital in Nadroga/Navosa Sub-division and it accepts primary referrals from its Health-Centers, while it refers cases to its tertiary hospital—Lautoka Hospital.

Purposive sampling was used, which included all the patients who attended SOPD at SSH during the study period. Potential sources of bias were addressed by attempting to reduce selection bias by allowing all eligible participants to be part of the study. Every participant who qualified under the study criteria was eligible for the study and patients were recruited every day during the study period. As a result, from a total of 265 patients who satisfied the inclusion/exclusion criteria, a sample size of 225 was finally selected to participate in this study.

### Measures

The data collection tool was the KAP questionnaire ( Mohammadnezhad, 2019), which had been developed by reviewing the literature and using other similar questionnaires that have been used previously, like the CKD Screening Index ( Khalil & Abdalrahim, 2014) and KAP questionnaire developed by Stanifer *et al.* (2015). This questionnaire was divided into two sections: Section A contained general information on 7 factors and Section B measured the KAP aspect of T2DM patients with CKD. Knowledge-related questions asked about signs/symptoms, prevention, treatment and risk factors. For the knowledge component, each item was given a score of “2” for a correct answer, “1” for “do not know” and “0” for incorrect response. Hence, the total scoring range for this section of 15 questions was 0–30 for each participant. Those with a score of 0–15 were considered as “low level of knowledge”, 16–22 as “medium level of knowledge” and 23 and over as “high level of knowledge” ( Kim, 2008; Tekanene *et al*., 2018). The attitude-related questions focused on the feelings of the participants based on the impact, effect, awareness and future implications. For the attitude component, each item was given a score of “2” for a positive attitude, “1” for “neutral” and “0” for negative attitude. Hence, the total scoring range for this section of 15 questions was 0–30 for each participant. Those with a score of 0–15 were considered as “low level of attitude”, 16–22 as “medium level of attitude” and 23 and over as “high level of attitude” ( Kim *et al*., 2009; Lincoln *et al*., 2018). The practice-related questions asked about self-care, management, control and behaviors. For the practice component, each item was given a score of “1” for a positive practice and “0” for negative practice. Hence, the total scoring range for this section of 10 questions was 0–10 for each participant. Those with a score of less than 5 were considered as “low level of practice” and 5 or over as “high level of practice” ( Kim *et al*., 2008; Tekanene *et al*., 2018).

Before collecting the data, face validity was assessed among 10 volunteer T2DM patients with CKD who were attending the SOPD clinic at SSH and satisfied the inclusion/exclusion criteria (5 males and 5 females) to assess whether the questionnaire was legible, clear, simple, easy and understandable. The content validity was also conducted by an expert panel to decide whether the content of the questionnaire met the objective of the study or not. Apart from the English version, the questionnaire (plus the information sheet and consent form ( Mohammadnezhad, 2019)) was translated into two other languages (Hindi and iTaukei) by a bi-lingual translator and then cross-translated to ensure the contents of the original questionnaire matched the translated version.

### Data collection process

After providing the information sheets, those who were eligible and consented for the study were given the questionnaire to either fill and return on the same day or return it later before the due-date by dropping it in the specially marked box in SOPD. The participants who were unable to complete the questionnaire on their own were assisted by the research assistant who provided non-bias support in filling the questionnaire on their behalf.

### Data analysis

All the questionnaires received by 31st August 2018 were used for analysis, while the rest were classified as non-responders. From a total of 249 questionnaires handed out, 232 were returned but only 225 were completely filled. The information from the questionnaire was entered in Microsoft Excel Data Sheet for cleaning and coding after which it was transferred to SPSS Version 25. The continuous variables were analyzed and expressed as means and standard deviation while the categorical variables were displayed as counts and percentages in a frequency distribution table. The Kolmogorov-Smirnov test was used to assess the normality for continuous variables. The tests of baseline differences in demographic characteristics and practice scores were done using independent t-test and ANOVA. Apart from comparing gender with KAP, all other comparisons were made using non-parametric tests to check the differences between demographic characteristics and knowledge and attitude respectively. Multiple linear regression analysis was conducted to see which independent variables were significant predictors of the dependent variable. P<0.05 was considered to indicate statistical significance.

### Ethical considerations

Ethical approval for this study was obtained from the Fiji National University College Health Research Ethics Committee (CHREC) and the Fiji National Health Research Ethics and Review Committee (FNHRERC) – approval number 2018.128.W.D. Each participant provided their written informed consent to take part in this study.

## Results

### Participant demographic characteristics

The study sample for this research comprised of 225 participants aged 38–92 years (mean=58.6, SD=9.99). Over half of the participants were in the age range of 46–60 years (52.4%) and there was almost an equal number of males (48.9%) and females (51.1%). In terms of ethnicity, there was almost an equal number of iTaukei (48.9%) and Fijian of Indian Descent (FID) (48.4%). In total, 48% of the study participants had secondary-level education, 38% of the participants were employed, 80.4% were married and 67.1% of the participants had an average monthly income of <$400 (see Table 1).

**Table 1.** Demographic Characteristics of Participants (n = 225).

Variables	Categories	N	%
Age (years)	30–45	18	8.0
	46–60	118	52.4
	61–75	75	33.3
	>76	14	6.3
Gender	Male	110	48.9
	Female	115	51.1
Ethnicity	iTaukei	110	48.9
	Fijians of Indian Descent	109	48.4
	Fijians of Others Descent	6	2.7
Level of Education	Uneducated	10	4.5
	Primary	75	33.3
	Secondary	108	48.0
	University	32	14.2
Employment Status	Unemployed	72	32.0
	Employed	86	38.2
	Domestic Duties	67	29.8
Marital Status	Single	16	7.1
	Married	181	80.4
	Divorced	6	2.7
	Widow	22	9.8
Average Monthly Income ($FJD)	<400	151	67.1
	401–800	40	17.8
	801–1200	18	8.0
	>1201	16	7.1

### Participant’s knowledge, attitude and practice

Table 2 displays the overall scores for the participant’s KAP. The highest score for knowledge was 30, while the mean score was 23.3 (SD ±3.25), which shows that the overall knowledge was high. For the attitude component, the highest score was 28 while the mean was 23.1 (±2.73) which show that the overall attitude was high. The highest score for practice was 10 while the mean was 7.1 (±2.04), which shows that the overall practice was also high.

**Table 2.** Overall scores of participant’s knowledge, attitude and practice.

Variables	Lowest score	Highest score	Mean (±SD)
Knowledge	11	30	23.3 (± 3.25)
Attitude	12	28	23.1 (± 2.73)
Practice	2	10	7.1 (± 2.04)

### Correlation of demographic characteristics and KAP

Table 3 shows that there was a significant association between level of education and knowledge (p<0.001), employment status and knowledge (p=0.05), average monthly income and knowledge (p=0.03), ethnicity and attitude (p=0.002) and ethnicity and practice (p=0.001).

**Table 3.** Correlation of demographic characteristics and KAP of T2DM patients with CKD (n=225).

Variable categories	N	Knowledge			Attitude			Practice		
		Mean	SD (±)	p-value	Mean	SD (±)	p-value	Mean	SD (±)	p-value
Age (years)				0.07 *			0.13 ***			0.72 **
30–45	18	23.9	2.11		23.4	2.06		7.0	2.09	
46–60	118	23.8	3.31		23.3	2.67		7.3	2.01	
61–70	75	22.8	2.95		22.9	2.96		7.1	2.11	
>70	14	21.6	4.50		21.8	2.39		6.6	1.95	
Gender				0.91 **			0.53 ***			0.73 **
Male	110	23.4	3.19		23.1	2.75		7.2	2.15	
Female	115	23.3	3.32		23.2	2.72		7.1	1.93	
Ethnicity				0.85 ***			0.002 ***			0.001 **
I-Taukei	110	23.3	3.18		23.7	2.69		6.7	2.15	
FID	109	23.4	3.34		22.5	2.70		7.6	1.82	
FoD	6	23.3	3.27		23.8	0.98		6.0	1.67	
Level of education				<0.001 ***			0.53 ***			0.25 **
Uneducated	10	23.5	2.80		23.6	2.76		6.8	2.82	
Primary	75	22.2	3.52		22.9	3.13		6.8	2.12	
Secondary	108	23.5	2.92		23.1	2.58		7.3	1.91	
University	32	25.3	2.81		23.6	2.19		7.5	2.00	
Employment status				**0.05** ***			0.88 ***			0.61 **
Unemployed	72	24.1	3.38		23.2	2.78		7.1	2.02	
Employed	86	23.1	2.86		23.2	2.58		7.0	2.19	
Domestic duties	67	22.7	3.44		22.9	2.87		7.3	1.86	
Marital status				0.86 ***			0.85 ***			0.18 **
Single	16	23.8	3.56		23.2	1.80		7.5	1.75	
Married	181	23.4	3.06		23.1	2.84		7.2	2.00	
Divorced and Widow	28	23.3	3.25		23.1	2.44		7.1	2.35	
Average monthly income				0.03 *			0.17 ***			0.82 **
<$400	151	22.9	3.33		22.9	2.78		7.1	2.12	
$401–$1200	58	24.2	3.01		23.6	2.73		7.1	1.88	
>$1201	16	24.2	2.59		23.6	1.89		7.4	1.97	

*Kruskal-Wallis test. **Parametric test. ***Non-parametric test.

### Multiple regression analysis

Table 4 shows that all the independent variables could predict only 6.7% of the total knowledge scores. Age categories of 61–70 (t = -0.664, p = 0.05) and >70 (t = -1.653, p = 0.01) and Ethnicity (FID: t = -3.287, p = 0.001) were significant predictors of overall attitude score. Those aged 61–70 years had a 0.53-point lower attitude score compared to other age categories (with other variables constant) while those aged >70 years had 1.78-point lower attitude score compared to other age categories (with other variables constant). Similarly, FID had a 1.5-point lower attitude score compared to other ethnic groups (holding other variables constant). All independent variables could predict only 2.9% of the total attitude scores (R ^2^ = 0.094, adjusted R ^2^ = 0.029). Finally, ethnicity (FID: t = 3.714, p < 0.001) was the only significant predictor of overall practice score. FID had a 1.03-point higher practice score compared to other ethnicities. All independent variables could predict only 6.1% of the total practice scores (R ^2^ = 0.123, adjusted R ^2^ = 0.061).

**Table 4.** Multiple linear regression tests on independent variables.

Variable	Knowledge			Attitude			Practice		
	B	t	p-value	B	T	p-value	B	t	p-value
Constant	23.24	13.318	0.00	24.913	16.655	0.000	6.892	6.261	0.000
Age, years (ref. 30–45)									
46–60	0.409	0.491	0.62	-0.069	-0.097	0.92	0.120	0.229	0.82
61–70	-0.127	-0.137	0.89	-0.528	-0.664	**0.05**	0.009	0.016	0.99
>70	-1.395	-1.108	0.27	-1.784	-1.653	**0.01**	-0.364	-0.458	0.65
Gender (ref. Male)									
Female	0.118	0.218	0.83	0.035	0.075	0.94	-0.241	-0.709	0.48
Ethnicity (ref. iTaukei)									
FID	0.355	0.806	0.42	-1.242	-3.287	**0.001**	1.033	3.714	**0.001**
FoD	-1.526	-1.104	0.27	-0.498	-0.420	0.68	-0.944	-1.083	0.280
Level of education (ref. uneducated)									
Primary	-1.487	-1.365	0.17	-1.045	-1.119	0.26	0.904	0.138	0.89
Secondary	-0.466	-0.419	0.68	-1.160	-1.218	0.23	0.746	1.064	0.29
University	1.203	0.945	0.35	-1.105	-1.012	0.31	1.348	1.678	0.09
Employment status (ref. unemployed)									
Employed	-0.205	-0.340	0.73	-0.303	-0.584	0.56	-0.715	-1.875	0.06
Domestic duties	-0.879	-1.335	0.18	-0.263	-0.466	0.64	0.062	0.149	0.88
Marital status (ref. single)									
Married	0.608	0.703	0.48	0.090	0.121	0.90	-0.199	-0.365	0.72
Divorced/widowed	0.046	0.044	0.97	0.631	0.698	0.49	-0.790	-1.187	0.24
Average monthly income (ref. <$400)									
$401–$1200	0.240	0.432	0.67	0.546	1.144	0.25	-0.018	-0.051	0.96
>$1201	0.432	0.482	0.63	0.934	1.214	0.23	0.210	0.371	0.71
R ^2^	0.129			0.094			0.123		
Adjusted R ^2^	0.067			0.029			0.061		

B = beta coefficient

## Discussion

Participants with higher levels of education, the unemployed and those with high monthly income had higher knowledge, FID had low attitude but high practice scores, and the higher age category had lower attitude scores.

This research had sought to identify the determinants of knowledge, attitude and practice towards causes, prevention, diagnosis, treatment and management in T2DM patients with CKD at SSH in 2018. Those aged 61–70 years had a 0.53-point lower attitude score (p=0.05) compared to other age categories, while those aged >70 years had 1.78 points lower attitude score (p=0.01) compared to those in other age categories. On the contrary, those aged >30 years were associated with having a good attitude in another study ( Yusoff *et al*., 2016). White *et al*. (2008) noted that people aged less than 60 years had better knowledge of kidney disease but nil discussion on association between age and attitude was mentioned by the authors. However, the association between increased education and knowledge is consistent with other studies. White *et al*. (2008) had also reported higher knowledge in participants with higher education which was supported by Khalil & Abdalrahim (2014); Stanifer *et al*. (2016) and Yusoff *et al*. (2016). This result makes sense since the higher the level of education, the more knowledge a person will have. Conversely, older age was linked to higher knowledge in another article from USA ( Ryder *et al*., 2013) while it was associated with high practice scores in the Jordan study ( Khalil & Abdalrahim, 2014).

Age is an independent variable that has been linked to various diseases in multiple studies; for instance, a rise in age is directly linked with an increased risk of cardiac events ( Canto *et al*., 2012). Age has also been associated with KAP of diabetes, but there are few studies which have tested and found significant links between age and KAP in individuals with diabetic kidney disease (DKD) ( Islam *et al*., 2014).

In terms of ethnicity, the current research showed that FID had significantly lower attitude scores but higher practice scores than iTaukei and Fijians of others Descent (FoD). This finding is evident in one of the aspects of practice at the daily SOPD clinics at SSH, since in Fiji’s SOPD clinics, the majority of the patients attending the clinic are FID, while iTaukei patients usually default their booked clinics and thus end up in making the majority of the numbers for NCD-related admissions. Kazley *et al*. (2015) had found poor knowledge scores in the African Americans but there was no mention of attitude or practice ( Kazley *et al*., 2015).

The link between ethnicity and health-related KAP is extremely important in the Pacific setting as they are usually culturally influenced and tend to prioritize behaviors practiced by their ancestors over the past generations—this means that if their ancestors had certain attitude or practice regarding a health-issue, the Pacific people are inclined to follow the same. Culture seems to play an integral part in lots of decision-making in these PICs, as preferences are governed by the ethnic roots of most of the Pacific Islanders ( Ryan *et al*., 2010). Subsequently, understanding and identifying the specific ethnic group with low KAP in SSH would help in tailoring the suitable health campaigns which are culturally appropriate and effective. Thus, this research shows that the iTaukei ethnicity will need to be considered while drafting and designing future public health preventive programs, as these are the individuals who need assistance with KAP towards DKD at SSH. Government could enact healthy lifestyle changes like compulsory tax on sugar, fat and tobacco and these legislative mechanisms can help the people of Fiji to be more inclined towards healthier choices, rather than the expensive, unhealthy ones.

The level of education had the strongest link with knowledge scores of the participants at SSH in 2018 whereby those with the highest level of education had higher mean knowledge scores. This result makes sense since the higher the level of education, the more knowledge a person will have. However, high education levels do not necessarily equate to high levels of attitude and practice ( Sa’adeh *et al*., 2018). White *et al*. (2008) had also reported higher knowledge in participants with higher education which was supported by Stanifer *et al*. (2016); Khalil & Abdalrahim (2014) and Yusoff *et al.* (2016).

Health literacy deals with an individual’s ability to obtain, read, process and understand health-related information to make applicable health decisions ( Jain & Green, 2016; Van den Broucke, 2014). The influence of health literacy on health-related decision-making helps to explain the link between knowledge and level of education as shown by this current study in SSH. As the participants of this study with higher levels of education are likely to have a better ability to comprehend medical information given to them, it seems likely that their knowledge scores would be relatively greater compared to those who have lower level of education (thus lower health literacy). Despite good self-reported ‘knowledge’ demonstrated in the survey, these were high-risk patients with a major complication and are at high risk for further poor outcomes. Thus, knowledge does not necessarily equate to good management at all times.

A major challenge for health professions is to use familial language (free of medical jargon) so that all at-risk populations with low health literacy levels understand how to best manage risk factors for diabetes-related complications. The real challenge of the health sector lies in this finding, since it means that health information must be translated into the simplest terms (free of medical jargon) and made available in the widest accessible form, so as to reach the greater subset of the population who lack higher education.

There are a lot of changes that need to occur in Fiji to reduce the epidemic of people developing diabetes and complications such as, improvement in patient awareness, patient-centered management, community-based active screening rather than passively waiting for patients to come for routine check-ups, and mobilizing community partnerships to reach out to the people on a larger scale.

Surprisingly, unemployed participants were found to have significantly higher level of knowledge in this study, although were not associated with better attitude and practice. Stanifer *et al*. (2016) had found similar link between unemployed participants and knowledge ( Stanifer *et al*., 2016). On the contrary, Yusoff *et al*. (2016) concluded that employed people had higher knowledge and attitude which was supported by Li *et al*. (2014) and Ryder *et al*. (2013). These are lot of contrasting findings regarding link between employment and level of knowledge and perhaps future studies of DKD patients locally, regionally or internationally could clarify the doubts.

Ruhm (2005) found that health-related prevention behaviors were higher in unemployed people. This analysis was made from a Behavioral Risk Factor Surveillance System (BRFSS) in USA, and could probably explain the findings of the current research whereby unemployed participants had higher knowledge levels ( Ruhm, 2005). However, the attitude and practice levels could not be linked with unemployment in SSH in 2018 and thus it is difficult to use the BRFSS solely as it deals with behaviors rather than knowledge alone.

Patients with T2DM and CKD at SSH in 2018 with high average monthly income had significantly higher knowledge levels regarding DKD, although the link to attitude and practice was insignificant. Similarly, Yusoff *et al*. (2016) found poor knowledge in those with low family income, while Khalil & Abdalrahim (2014) had showed that participants with high monthly income had high practice scores. Income is one of the key pillars of socioeconomic status (SES)—the other two being occupation and education. Therefore, it makes sense if participants with high monthly income have high knowledge since the interplay between the social gradient and health literacy is quite predictable ( Diamond *et al*., 2011; Quinlan *et al*., 2013).

Fiscella (2016) stated that a person’s behavior is limited by their SES and hence their health-seeking behavior will change if their access to resources is increased ( Fiscella, 2016). This means that the socio-economic disparities of participants with lower monthly income at SSH will need to be tackled by primary healthcare workers to influence KAP. This is difficult and thus may involve health and policy-making at the operational level to take into account the issues of health equality and equity.

### Strengths and limitations of the study

The strengths of the study are that this is probably the first study done in Fiji to focus on the KAP of patients with T2DM and CKD. The baseline demographical information showed almost equal representation of sample in terms of gender, ethnicity and employment status, and thus the selection biasness is substantially reduced. The availability of the survey tool in three languages enabled the collection of data from all the ethnic groups (FID, iTaukei and FoD).

The strengths of the study are that this is probably the first study conducted in Fiji to focus on the KAP of patients with T2DM and CKD. Despite the study sampling patients attending health clinics during the working week, the baseline demographical information showed almost equal representation of sample in terms of gender, ethnicity and employment status, and thus the reducing the potential for selection biasness is substantially reduced. The availability of the survey tool in three languages enabled the collection of data from all the ethnic groups (FID, iTaukei and FoD). Limitations of this study include the moderately small sample size and thus there may have been greater or more significant inability to detect small differences between groups. Due to the questionnaire being self-answered administered by the participants, there is also a high chance the potential for incorrect responses information bias of errors or misrepresentation of information due to chance of information with a bias toward ‘politically correct’ responses. Finally, there were no associations measured between knowledge, attitude, behaviors and risk factors. Normative reference groups were used for the multiple regression analysis regardless of numbers in each group.

## Conclusion

This research was able to identify high-risk groups with low levels of knowledge, attitude and practice, towards whom targeted public health interventions can be targeted in future. The results of this study can enable informed public-health policies to be made to target the high risk specific groups with low KAP scores and consequently potentially increasing their knowledge, attitudes and practice within these populations through well-planned, appropriate and tailored strategies that are culturally acceptable and suit the identified groups. Consequently, health promotion activities are vital in improving knowledge, attitude and practice, and it is recommended that health promotion and interventional studies to be conducted using the results of this study among patients targeted toward at-risk groups identified in this study to potentiate the greatest improvements in health outcomes and to measure the effectiveness of health promotion intervention. Further research like clustered randomized intervention trials are necessary to identify test strategies to improve management and outcomes for these high risk patients.

## Recommendations

It is recommended that in order to raise the level of knowledge, attitude and practice, in high risk populations, the barriers to healthcare need to be identified and removed or reduced. There could be for example, provisions made for mobile outreach health-clinics for rural communities, who have difficulty in accessing hospital services. Additionally, there needs to be financial support or free transport for patients to attend clinic, if they face difficulties in accessing medical care due to financial issues. The patients need to have convenient and varied clinic times that include pharmaceutical services. They need to be booked for clinics at a time suitable for them keeping in mind their transport issues, distance of travel, work and other commitments, and so forth. Thus, flexibility in the appointment system for patients can help increase their compliance to attendance at clinics (including mobile clinics) therefore increase their access to wide array of health services thus leading to an increase the number of contacts with health professional, improve self-management for those with chronic conditions, reduce diabetes-related complications and reduce inequalities in health care provision in Fiji and greater medical services including awareness. The barriers to healthcare need to be identified and consequently attempts made to reduce them so as to help empower patients to live a healthier life, make smart health choices, manage their illness and avoid complications. The most important factor will be to recognize the high risk patients so that an intensive and comprehensive health care intervention/program is delivered to them.

## Data availability

### Underlying data

Open Science Framework: T2DM among CKD patients. https://doi.org/10.17605/OSF.IO/A25GD ( Mohammadnezhad, 2019). Raw data are included in the indicated file.

### Extended data

Open Science Framework: T2DM among CKD patients. https://doi.org/10.17605/OSF.IO/A25GD ( Mohammadnezhad, 2019).

This project contains the following extended data:

- Questionnaire.pdf (questionnaire in each language).
- Consent Forms.pdf (in each language).
- Information sheet.pdf (information for participants in each language).

Data are available under the terms of the Creative Commons Zero “No rights reserved” data waiver (CC0 1.0 Public domain dedication).
