# Supplementary material for: Determinants of knowledge, attitude and practice in patients with both type 2 diabetes and chronic kidney disease in Fiji
Source: F1000Res. 2019 May 1;8:239. Originally published 2019 Mar 1. [Version 3] doi: 10.12688/f1000research.18188.3 (PMC6480935; doi:10.12688/f1000research.18188.3)
Supplement: Peer review history

# Peer review: Reviewer response for version 2

**DOI:** 10.5256/f1000research.20722.r45136

**Published:** 2019-04-23

**Authors:** Sharyn Burns

**Affiliations:** 1 Collaboration for Evidence, Research and Impact in Public Health (CERIPH), School of Public Health, Curtin University, Perth, WA, Australia

This is an interesting and relevant paper. The paper is well constructed. More information about the items that comprised the knowledge, attitudes and practice scores should be included in the methods. While it notes the questionnaire is available as a supplement it is difficult for the reader to discern what the key focus of the KAP questions are. A summary of the focus, and some examples of the types of questions asked would be useful.

 The conclusion of the abstract identified there were some high risk groups identified – it would be useful to state which groups these were.

 In the paragraph commencing Ruhm (2005) found … (in the Discussion) the study found unemployed participants to have higher knowledge levels. Did you measure if participants were retired? As there is a correlation between education and knowledge there may be some correlation between well educational, but retired participants? It would also be useful for the reader to have some background to unemployment in Fiji – is unemployment high? Did you collect data about unpaid work (eg. parents that may care for children etc).

 The paper needs editing. Sentence composition could be improved. I have highlighted some suggested edits, however an overall edit is recommended.

**Edits:**

Abstract:

 ‘Spearman correlation and multiple linear regressions were also done’.

 Rather than ‘were done’ sounds better to say ‘were conducted’

 ‘All the tests were set at 5% level of significance’

 Delete ‘the’ eg “all tests …”

 Check sentence composition: ‘the ninth-leading cause of deaths globally (Rifkin *et al*., 2012), is defined as estimated glomerular filtration rate (eGFR) of less than 60 ml/min for at least 3 months’

 Does the following sentence relate to the adult population: ‘Fiji’s STEP-wise Surveillance Report (STEPS) of 2011 showed that 15.6% of the population had raised  fasting blood sugar’

 ‘This is quite worrying’; it is better to say “this is concerning ..”

 ‘hence trimming these expenses via addressing these 2 diseases from a public health perspective would be beneficial’: This sentence needs clarifying – what does ‘trimming these expenses’ refer to?

 ‘which uses questionnaires’: change to ‘uses scales and items to …’

 ‘while married ones had high attitude and practice’: change to ‘while married respondents reported high …’

 Avoid using terms like ‘on that same note ..’ and ‘on the other hand’ throughout the paper

Discussion:

 ‘but nil association with attitude was mentioned’: I am not sure what ‘nil association’ refers to

 ‘These are lot of conflicting information regarding employment and level of knowledge’: this sentence composition needs work

I have read this submission. I believe that I have an appropriate level of expertise to confirm that it is of an acceptable scientific standard.

# Response

**Published:** 2019-04-24

**Authors:** Masoud Mohammadnezhad

**Affiliations:** Fiji National University, Fiji

Abstract:

 1- ‘Spearman correlation and multiple linear regressions were also done’. Rather than ‘were done’ sounds better to say ‘were conducted’

 Changed as per reviewer’s comments

 2- ‘All the tests were set at 5% level of significance’ Delete ‘the’ eg “all tests …”

 Changed as per reviewer’s comments

 3- Check sentence composition: ‘the ninth leading cause of deaths globally (Rifkin ., 2012), is defined et al as estimated glomerular filtration rate (eGFR) of less than 60 ml/min for at least 3 months’  

 Sentence composition restructured

 4- Does the following sentence relate to the adult population: ‘Fiji’s STEP wise Surveillance Report (STEPS) of 2011 showed that 15.6% of the population had raised fasting blood sugar’

 Yes, it refers to adults in the age-range of 25-64 years old – this has been added to the main text

 5- ‘This is quite worrying’; it is better to say “this is concerning ..”

 This phrase was removed after first review

 6- ‘hence trimming these expenses via addressing these 2 diseases from a public health perspective would be beneficial’: This sentence needs clarifying  what does ‘trimming these expenses’ refer to? 

 Clarification done

 7- ‘which uses questionnaires’: change to ‘uses scales and items to …’

  Changed as per reviewer’s comments

 8- ‘while married ones had high attitude and practice’: change to ‘while married respondents reported high

 Changed as per reviewer’s comments

 9- Avoid using terms like ‘on that same note ..’ and ‘on the other hand’ throughout the paper  

 Changed as per reviewer’s comments

Discussion:

 10- ‘but nil association with attitude was mentioned’: I am not sure what ‘nil association’ refers to  

 The authors of the paper had not tested or discussed association between age and attitude score – added to main text.

 11- ‘These are lot of conflicting information regarding employment and level of knowledge’: this sentence composition needs work

 Sentence structure changed.

# Peer review: Reviewer response for version 2

**DOI:** 10.5256/f1000research.20722.r47311

**Published:** 2019-04-23

**Authors:** Barbara M. Daly

**Affiliations:** 1 School of Nursing, Faculty of Medical and Health Sciences, University of Auckland, Auckland, New Zealand

Authors have made changes in line with the first review and feedback. I particularly commend the authors on changes regarding culture and the role of Government in potentially changing policy to reduce the burden of diabetes and kidney disease. However, I note that reference groups for multiple regression were not addressed. See my suggestion for addressing this in the limitation section. Several edits are required – I have outlined these with suggestions below.

Results -Table 4 multiple regression

 I note that you have not changed the reference groups to those with greater numbers. In the limitation section can you acknowledge this and justify why these reference groups were used please. See my suggestion below under ‘limitations’.

Edits required

 1. Remove stop after panel.

 The content validity was also conducted by an expert panel. To decide whether the content of the questionnaire met the objective of the study or not.

 2. Discussion – this phrase is not clear – do you mean ‘no association with attitude was found’

 but nil association with attitude was mentioned.

 3. I suggest deleting the sentence below and add this into the last sentence of this paragraph.

 ‘However the association between increased education and knowledge is consistent with other studies White *et al*. (2008) had also reported higher knowledge in subjects with higher education which was supported by Stanifer *et al*. (2016); Khalil & Abdalrahim (2014) and Yusoff *et al.* (2016).

 This result makes sense since the higher the level of education, the more knowledge a person will have.

 4. Please change ‘subjects’ to participants.

 5. Please remove space after ‘self-…

  Despite good self- reported ‘knowledge’

 6. The sentence below is not clear – I suggest the following ‘A major challenge for health professions is to use familial language (free of medical jargon) so that all at-risk populations with low health literacy levels understand how to best manage risk factors for diabetes-related complications.

 The real challenge of the health sector lies in this finding, since it means that health information must be translated into the simplest terms (free of medical jargon) and made available in the widest accessible form, so as to reach the greater subset of the population who lack higher education.

 7. Grammar – please change to  ‘There is much conflicting information…

 These are lot of conflicting information

 8. Limitations - See suggested changed below in limitations.

 The strengths of the study are that this is probably the first study done in This is the first study conducted in Fiji to focus on the KAP of patients with T2DM and CKD. Despite the study sampling patients attending health clinics during the working week, the baseline demographical information showed almost equal representation of sample in terms of gender, ethnicity and employment status, and thus the reducing the potential for selection biasness is substantially reduced. The availability of the survey tool in three languages enabled the collection of data from all the ethnic groups (FID, iTaukei and FoD).

 Limitations of this study include the moderately small sample size, was very small and thus there may have been greater or more significant limiting the ability to detect small differences between groups. if sample size was large. Due to the questionnaire being self-answered administered by the participants, there is also a high chance the potential for incorrect responses information bias of errors or misrepresentation of information due to chance of information with a bias toward ‘politically correct’ responses. Finally, there were no associations measured between knowledge, attitude, behaviors and risk factors. Normative reference groups were used for the multiple regression analysis regardless of numbers in each group.

 9. Conclusion and Recommendations – see suggested changes below.

 Conclusion

 This research was able to identify Results from this study identified high-risk groups with low levels of knowledge, attitude and practice, towards whom for targeted public health interventions can be targeted in future. The results of this study can enable In addition, findings can inform informed public-health policies to be made to target the for high risk groups specific groups with low KAP scores and consequently potentially increasing their knowledge, attitudes and practice within these populations through well-planned, appropriate and tailored strategies that are culturally acceptable suit the identified groups. Consequently, health promotion activities are vital in improving knowledge, attitude and practice, and it is recommended for that health promotion and interventional studies to be are conducted using the results of this study among patients are targeted toward at-risk groups identified in this study to potentiate the greatest improvements in health outcomes. to measure the effectiveness of health promotion intervention. Further research is Clustered randomised intervention trials are necessary to identify test strategies to improve management and outcomes for these high risk patients.

 10. Recommendations

 It is recommended that in order to raise the level of knowledge, attitude and practice, in high risk populations, the barriers to healthcare should be need to be identified and removed, reduced. There could be for example, provisions made for provision of mobile outreach health-clinics for rural communities, who have difficulty in accessing hospital services. Additionally, there needs to be financial support or free transport for patients to attend clinic, if they face difficulties in accessing medical care due to financial issues. The patients need to be and convenient and varied clinic times that includes pharmaceutical services. booked for clinics at a time suitable for them keeping in mind their transport issues, distance of travel, work and other commitments, and so forth. Thus, flexibility in the appointment system for patients can help increase their These measures need may would be expected to increase compliance to attendance at clinics (including mobile clinics) therefore increase their access to wide array of leading to an increase the number of contacts with health professional, improve self-management for those with chronic conditions, reduce diabetes-related complications and reduce inequalities in health care provision in Fiji and greater medical services including awareness. The barriers to healthcare need to be identified and consequently attempts made to reduce them so as to help empower patients to live a healthier life, make smart health choices, manage their illness and avoid complications. The most important factor will be to recognize the high risk patients so that an intensive and comprehensive health care intervention/program is delivered to them.

I have read this submission. I believe that I have an appropriate level of expertise to confirm that it is of an acceptable scientific standard.

# Peer review: Reviewer response for version 2

**DOI:** 10.5256/f1000research.20722.r47312

**Published:** 2019-04-17

**Authors:** Gade Waqa

**Affiliations:** 1 Fiji National University, Suva, Fiji

I have read this submission. I believe that I have an appropriate level of expertise to confirm that it is of an acceptable scientific standard.

# Peer review: Reviewer response for version 1

**DOI:** 10.5256/f1000research.19895.r45138

**Published:** 2019-04-02

**Authors:** Barbara M. Daly

**Affiliations:** 1 School of Nursing, Faculty of Medical and Health Sciences, University of Auckland, Auckland, New Zealand

**General feedback**

 This paper reports on knowledge, attitudes and practice or behaviours of diabetes patients with chronic kidney disease (CKD) surveyed while attending an out-patients clinic in Suva, Fiji in 2018. Despite patients having a major complication of diabetes, indicating poor control of HbA1c, hypertension and tobacco use, overall patients displayed a good knowledge, attitude and behavioural practice toward diabetes. This may reflect increased knowledge and practice in long-term diabetes patients who have to manage a serious complication or it may reflect a disconnect between knowledge and good self-management.

 Overall, authors identify factors that relate to knowledge, attitudes and practice that may influence health behaviour and outcomes in diabetes patients with CKD. However, authors need to carefully edit this manuscript, greatly reduce the word count by deleting repetitive themes, ensure results are clear, review reference groups for multiple linear regression analyses and adhere to the usual academic writing style. Specific suggestions to improve the paper are outlined below.

**Title **– suggest 'Determinants of knowledge, attitude and practice in patients with type 2 diabetes and chronic kidney disease in Fiji'

**Abstract:**

**Methods**

- Only refer to Fiji and elaborate later, only include the year and self-administered questionnaire.
- You need to include the number of patients / total who came to the clinical and number in study.
- Avoid most abbreviations in the abstract.
- Remove unnecessary descriptions: name of hospital (Fijian citizens, special out-patients department, (Suva, Fiji is sufficient).
- Remove most of the statistical information and leave this to methods section.

 
**Results **

- Outline numbers in study
- Response rate – this is very important
- Results:  Out of what score i.e. 23.3 / 30.
- Can you qualify ‘unemployed’. For example, are these mostly students, mothers are they collecting an unemployment benefit, are they actively seeking work.  As it stands this large group of participants do not typically resemble ‘unemployed people’ (around 5% in developed countries at the moment. Explain why so many people in the study were ‘unemployed’. This will help you contextualise unemployed group surveyed.

 
**Conclusion**

- Summarise the main outcomes are rather than KAP so the reader fully understands this and avoid just repeating the results.

**Introduction**

- Please give some more details about diabetes in Fiji – almost all have type 2 I believe. What proportion have CKD (what proportion are on dialysis and is this available to al. What is the mortality rate for patients with CKD. 
- If you cannot find national data can you look at the data from the clinic? If not you need to suggest that this is done in a ‘recommendation section’ in the discussion. It is important to give readers a sense of the seriousness of the epidemic in Pacific countries.

Grammar

- Remove first sentence
- Delete all emotive language i.e. ‘this is quite worrying’ instead compare 15.6% with other populations.
- Paragraph two – check description. When was this developed and is it considered the best tool to test these attributes. Is there evidence to suggest that patients who score highly are more likely to have fewer complications? Has this been used before in high-risk patients (diabetes and CKD)?
- Paragraph 3 – change beginning to: This is the first study in Fiji …

 
**Methods**

 Sample – it is important to describe this. What number of patients registered at the clinical with diabetes and CKD? Look at your sample does this represent the gender, ages, ethnicity, education etc. of the total outpatient clinic – this is what you need to discuss regarding selection bias. If Polynesian Fijians are less likely to come for appointments, are they underrepresented?  Also comment on this in the limitation section especially if you cannot get these details about the total cohort of patients registered.  If you cannot get data on the total cohort i.e. to check gender, age and ethnicity then you need to discuss whether the patients sampled are representative of all patients registered or enrolled in the clinic.  Are all patients in Suva able to register – what about rural based patients?

Unemployed – I suggest a different definition.

 It important you give more information about ‘unemployed’ because your finding do not fit the usual picture.  A third of your sample are unemployed. How was this defined i.e. on a benefit, actively looking for work and what is the unemployment rate in Fiji. I wonder if a large proportion of these participants are students or recent graduates i.e. well educated but not employed. Are better-off family members or partners supporting some of them? If many are in the younger age categories this might be the case so you could have a look at the data. Alternatively, are they too sick to work? If Fiji has a very high unemployment rate then you need to explain this so that the reader knows that being unemployed is different to the usual characteristics of most unemployed people receiving a benefit. If there is a strong other characteristic such as recent university graduate then call this something else i.e. currently seeking employment or something that better defines them.  It is very confusing for readers as this group are atypical. Are they largely mothers who have not returned to the workforce?

 Please briefly define how ‘knowledge, attitude and practice’ were measured and explain the main focus of the knowledge questionnaire i.e. mostly about diabetes and HbA1c control and consider including a couple of key questions. What were the main ‘attitude questions’ i.e. positive attitude, negative (explain to the reader) and similarly with practice i.e. include behaviours measured such as smoking. Consider given a couple of examples to illustrate the focus. 

- Bring the information about the questionnaire into this section. Also, cover the history of the questionnaire.
- Remove unnecessary text i.e. SPOD – only include descriptions if this is necessary.
- Remove ‘most importantly’ – no one will participate if they don’t want to. Did they sign a consent form or give oral consent. We know you cannot make patients participant. What did you do to encourage patients to participate i.e. what measures to increase participation?
- Review exclusion criteria and avoid repeating ideas. What was mental ability i.e. an inability to complete the questionnaire i.e. be specific.  It seems to me as if all patients with type 2 and CKD were invited to participate. However XX patients were unable to ‘read’, write or complete the questionnaire with assistance and were therefore excluded from the study. (I think only people who were incapable of completing the questionnaire were excluded so make this clear (this is good as most people were able to participate).
- Para 2 – review and delete as most of this is repetition
- Para 3 – describe accurately i.e. all patients who attended … see above most of this is repetition.
- A cross-sectional study is quantitative not convenient. Describe the cohort of patients eligible, were patients recruited every day during the study period. If all patients were invited, provided they could complete the questionnaire, then state this. Move comment on bias to the discussion under strengths and limitations.
- Para 4 – briefly state the total score in the abstract as this is important.
- Para 5 – Reduce this paragraph i.e. … the questionnaire was piloted by… to ensure face validity. Content validity was assessed by ?? an expert panel…  (you do not need all the details that you would include in a thesis).
- Data collection: reduce details and write succinctly. I suggest you change ‘illiterate’ to ‘unable to complete the questionnaire on their own’ i.e. they may need glasses to read!
- Data analysis – how many questionnaires were handed out how many returned? 

**Results**

 Titles – You have far too many titles so need to reduce the number.

- ‘Participant’ demographic characteristics. 
- Factors related to knowledge etc. 

Para 1

- Avoid repeating all data from the tables – summarise i.e. over half the patients were aged 46-60 years.
- Avoid repeating phrases i.e. participant in this study
- Define intake (indigenous or Polynesian – what do people prefer)
- Do not use FID (think about the international reader) Fijian Indian / Fijian of Indian decent or the official term or what people prefer.

 

Para 2 and 3

 Avoid repeating results. Summarise the main points.

Table 4

- Go through and review all your reference groups – these should be groups with a reasonable number of people not extreme groups with few numbers i.e. use 46-60 for age, level of education use secondary, use employed, use married, income either <400 or 4-800.
- Abbreviations should be listed under the tables B = beta coefficient 

Para 4

- Summarise the main points i.e. what factors were associated with knowledge … what were negatively associated. Similarly for attitude and practice
- Summarise overall findings – were any factors positively associated with all three attributes, two or none.
- Adjust text after you change the reference groups and reanalysis this data. 

**Discussion**

New Para 1

- Always write a summary of main findings a little like the above paragraph and use knowledge,
- attitude and practice. Readers need to be able to easily understand what was measured (avoid KAP in the main summaries).
- Are there any trends (see Para 4 above).
- You did not measure prevention. All of these patient were already diagnosed and have a major complication (CKD). Be specific to this group of patients sampled. You cannot generalise your results to other diabetes patients. 

New para 2

- Comparing main results with other study findings. Typically cite the studies with same findings then studies with opposite findings.
- Avoid informal phrases i.e.  ‘another article from the USA’ … 

Old Para 2

- Delete first sentence this is irrelevant.
- Focus on Type 2 diabetes with major complications i.e. CKD.
- CKD is related to time since diagnosis and exposure to risk factors and other factors such as genetics, ethnicity. 

Para 3

- Move this to the methods section and describe the total cohort of patients with type 2 and CKD registered at the outpatients clinic.
- Delete all judgemental statements. 
- Include barriers to good health care facility in Fiji and cite evidence.  
- Include a recommendation section at the end. Include recommendations to reduce barriers to healthcare i.e. mobile clinic for rural-based patients, funding so they can attend clinic, free transport (buses to clinic), convenient time for appointments. Think about why patients do not come to appointments. 

Para 4

- Review this para. You are being very judgemental and have very few references. Please appreciate that all Pacific populations have been influenced by unhealthy western lifestyle changes. When you think about it if Pacific populations continued the behaviour of their ancestors they would be fit, lean, non-smoking and very physically active.
- Consider how could the Government could enact healthy lifestyle changes i.e. tax on sugar, fat and tobacco?
- Avoid comparing ethnic groups unless you have good evidence.
- Focus on your findings on knowledge, my understanding is that this was not to ethnicity! 

Para 5

- Move this into comparisons with other studies above. 

Para 6

- You did not measure health literacy you cannot bring this into your findings. Focus on the associations you found.
- Remember that you found a good mean level of knowledge and yet these patients had progressive complications for diabetes. Knowledge does not always equate to  better management.  Highlight that despite good self-reported ‘knowledge’ demonstrated in the survey these were high-risk patients, with a major complication and are at high risk for further poor outcomes. 
- Can you comment on what changes need to occur in Fiji to reduce the epidemic of people developing diabetes and complications?

Para 7

- Review definition of ‘unemployed’. This usually refers to people receiving an unemployment benefit and looking for work. A third of the sample are ‘unemployed’ so it is very different from the usual unemployed group in developed countries.
- Again focus on your results and comparative studies. Avoid discussing other issues that you did not examine in your survey. 

Para 8

- This is not clear.  Do not bring in other questionnaires but refer to other studies that support your findings or contrast and try to explain why there might be differences between your sample and other populations.
- Unemployment in the US is very different from in Fiji i.e.  <5% are unemployed in the US. 

**Limitations**

- Discuss the questionnaire. Is it an ideal questionnaire to test knowledge, attitudes and practice in the high-risk group of patients who already have CKD. Are other populations where this questionnaire has been tested the same as people registered in the outpatients in Suva? If not how might the questionnaire have been adapted. Were there knowledge, attitude and practice questions about CKD and management of risk factors?
- My understanding is that the questionnaire focused on Hba1c. I believe there were very few questions about other risk factors such a hypertension, smoking and cholesterol which are very important for good management and to avoid further renal dysfunction. Point this out as a limitation of the survey tool or questionnaire. Recommend that the questionnaire may need updating to include all risk factors for complications and adapted for those with progressive serious complications. This tool may not be a good measure of healthy behaviour in diabetes patients with CKD i.e. has it been used in this group before i.e. very high-risk group of people with type 2. This is important for other researchers to consider.
- You must compare you sample with the total cohort of high risk patients in the clinic not just those in the sample (age ethnicity and gender). If your sample differs from the whole cohort then it is not representative of the total cohort.
- You must be specific when discussing bias and why (which way will it increase bias) i.e. selection, information and confounding. Be clear which potential bias you are referring to.
- You must say why the sample size was small i.e. would there be greater differences or significant differences if the sample was larger.
- Why do you say this is not generalizable?  Provide you asked all diabetes patients with CKD over the study period it will be representative of those high risk patients during the study period provided equal proportions of people not come to their clinic appointments. If this was the case you need to state this provided you have data on this.
- Comment on self-reported responses and the implications of this i.e. information bias toward ‘politically correct’ responses. Because this is not random error information bias in this case information bias would be in the positive direction.
- No clinical indices were measured. For example you do not report Hba1c so you cannot report on associations between knowledge attitude, behaviours and risk factors.

New Recommendation paragraph

- See suggestions above.
- Mostly to reduce barriers to health care, support for patients to improve lifestyles and to better manage risk factors for complications. Most important how to improve healthy behaviours in this high risk patients group – they need intensive health care provision.

 
**Conclusion **

- Summarise the main findings. Delete all data and avoid using KAP.
- Move the last paragraph up to the first under conclusion and focus on the main findings.
- You cannot make most of these statements as you do not know what those strategies are. 
- I suggest you finish on how on further research is necessary to identify strategies to improve management and outcomes for these high risk patients.

 
**Editing required**

- Delete all emotive language and lay descriptions i.e. on the other hand, it did not come as a surprise, this is not an easy task, it makes sense… check the whole document and delete all of these phrases. You need to write in standard academic language.
- Check all abbreviations and avoid non-standard abbreviations.
- Be consistent with people in the study either use ‘participants or patients – not subjects!
- Check titles and avoid unnecessary descriptors i.e. type two diabetes with CKF (we know this is the patient group).
-  Avoid repeating participants in study. You often have this twice in one sentence or paragraph.
- Check capitalisation i.e. ethnicity
- Most authors now use ‘among’ rather than amongst.
- Abbreviations should be listed under the tables ‘B’ = beta coefficient.
- Delete all judgemental statements.

I have read this submission. I believe that I have an appropriate level of expertise to confirm that it is of an acceptable scientific standard, however I have significant reservations, as outlined above.

# Response

**Published:** 2019-04-09

**Authors:** Masoud Mohammadnezhad

**Affiliations:** Fiji National University, Fiji

Thanks for your time and comments. Your comments were very useful and we tried to revise our paper based on your valuable comments. Kindly pleas find blow our response to your comments.

**Abstract**

1. It is better to clearly mention the setting of the study
2. The number mentioned (225) is based on the 2 months period of study for total eligible sample size – our sample size is not based on the total number of attendance
3. The abbreviations are based on the first repeat in the article and it already was reviewed by the journal editors
4. They all are study inclusion/exclusion criteria which need to be mentioned in the study method
5. A brief description of the tests used for this study is mentioned and we followed the word limitation of the journal

Results

1. Number of participants added
2. Sentence added to abstract about response rate
3. Score added
4. Employment status was categorized only based on yes/no and further details were not asked

Conclusion

1. Conclusion corrected as per reviewer’s comments

**Introduction**

1. Because of limited information, we have included all data that we could access and hence we do not have information about types of Diabetes in Fiji and the CKD dialysis and mortality rates in Fiji
2. Clinic data has not been audited and thus cannot be used for this study

Grammar

1. We have to define the terms used in this study
2. Emotive language changed as per reviewer comments
3. Since this is a quantitative KAP survey, a KAP questionnaire which has been developed from 2 KAP questionnaires (with Cronbach’s alpha of more than 0.7), this is the best tool for the KAP measurement. We are not doing a study to check association between KAP score and its link to complications of T2DM
4. Changed as per reviewer

**Method**

1. The hospital used for this study is a referral hospital and thus it receives patients from all settings, ethnicities, ages, gender, etc. This study showed that the ethnic distribution was almost equal in the 2 major ethnicities thus chance of biasness is limited.
2. The questionnaire did not ask details about employment status. This study did not look at the younger age categories thus they could not be recent graduates. The participants are attending SOPD clinic and usually are older age groups.
3. More info about the KAP questions added.
4. The information details were provided. Questionnaire history already mentioned in introduction
5. SOPD needs to be mentioned as abbreviation of the clinic.
6. Most-importantly removed. The details of recruitment is mentioned in data collection process and all patients were provided consent forms and information sheets. No incentives was provided for participation but the potential benefit of participation was provided in info sheet and hence they were happy to help researcher with the study.
7. Changed as per reviewer comments
8. Para 2 – since this is a description of study setting, it cannot be deleted entirely.
9. Para 3 – corrected
10. Corrected as per comments
11. Para 4 – already added total score
12. Para 5 – corrected
13. Data collection – corrected
14. Data analysis – corrected

**Results **

1. Titles – corrected
2. Para 1 – corrected
3. Para 2 and 3 – these are main finding hence need to be explained in text.
4. Table 4 – reference groups have been already identified and do not affect results. Abbreviation added
5. Para 4 – Main points summarized

**Discussion**

1. New para 1 added
2. New para 2 – not added but old para 1 revised
3. Old para 2 – first sentence not removed since it is used to explain the paragraph. Diabetic kidney disease is used interchangably for T2DM patients with CKD.
4. Para 3 – not moved since it is used to discuss the burden of CKD at SSH. The total cohort of patients with type 2 and CKD registered at the outpatients clinic at SSH is not available as there is no proper registration done. The comparison of FID is not a judgmental statement but others changed. Recommendation added in the end.
5. Para 4 – reviewed. Judgmental statements altered. Government statements added. Ethnicity used to show determinants of KAP hence it is used in discussion.
6. Para 5 – comparison is done in this paragraph since it follows the original pattern of discussion.
7. Health literacy mentioned to elaborate on the link between patient’s ability to understand/comprehend information and their level of knowledge as a consequence. However, knowledge part added as per reviewer comments. Fiji’s changes added as per comments.
8. Para 7 – unemployment details were not asked further in the main questionnaire hence cannot change it now. Other issues have been discussed despite not being examined in this study since they help to connect the points that have been identified in this research.
9. Para 8 – The other questionnaire is a reference to discuss the topic in the paragraph. Sample differences have been explained in the latter paragraphs.

**Limitation**

1. The questionnaire was ideal as its validity and reliability have been mentioned.  The other settings where KAP questionnaire have been tested are developing countries similar to Fiji. The KAP questions looked at both T2DM and CKD.
2. The other risk factors were not included in the questionnaire since the focus was only T2DM and its link to CKD. Additionally, T2DM is the major cause of CKD thus this link was tested in the questionnaire. Other behavioral risk factors were not deliberated.
3. It is difficult to compare my sample with the other cohorts in the clinic since there is no register to show the registered cases of high risk. The paper-based record keeping used in the clinic used for this study does not have any specific book to record all its cases by diagnosis and so forth.
4. Biasness explained
5. Sample size explanation added
6. Generalizability portion edited
7. Information bias added as per comment
8. Comment added to limitation

**Conclusion**

1. Main findings summarized. Changes made as per comments
2. Last paragraph moved to first
3. Statements revised
4. Changes made

**Recommendation**

1. New paragraph on recommendation added

# Response

**Published:** 2019-04-24

**Authors:** Masoud Mohammadnezhad

**Affiliations:** Fiji National University, Fiji

Thanks you for your comments and suggestions. We corrected as per your comments. We also changed the sentence structure as you advised.

# Peer review: Reviewer response for version 1

**DOI:** 10.5256/f1000research.19895.r45137

**Published:** 2019-03-27

**Authors:** Gade Waqa

**Affiliations:** 1 Fiji National University, Suva, Fiji

The authors utilized a self-structured questionnaire to test KAP of 225 patients at Sigatoka Sub-divisional Hospital in Fiji, and used independent t-test and ANOVA to test the differences between demographic variable and practical score while non-parametric tests were used for knowledge and attitude which allowed guidance to promote effective public health interventions to high risk groups. The power of using the survey tool in three languages was demonstrated through the high response rate, although the sample size was very small that lack the generalization of the results to all the population.

 It would be interesting in the discussion to compare this findings to any Fiji findings with other low income countries, including in the region if that is available.

 Clarification is needed regarding definitions of the three ethnic groups since the results are discussed using the different ethnicity category, as usually data for Fiji is based on three groupings.

 The method and discussion both mention the problem of poor knowledge and education level affecting data, yet the issue is not really explored as to its possible impact on the outcomes of the analysis. What are the implications of this? This should be discussed in the discussion section.

 The discussion focuses only on ethnic differences with KAP, would it be possible to also discuss the gender differences with KAP? It is interesting that many aspects of lifestyle are better in females than males according to STEPS 2002, and this should be discussed.

I have read this submission. I believe that I have an appropriate level of expertise to confirm that it is of an acceptable scientific standard.

# Response

**Published:** 2019-04-09

**Authors:** Masoud Mohammadnezhad

**Affiliations:** Fiji National University, Fiji

Thanks for your time and comments. I would like to respond to your comments as below:

1. No other similar KAP studies on T2DM patients with CKD was found that was done in other countries in the Western Pacific Region
2. The 3 ethnic groups used are the I-Taukei, Fijians of Indian Descent and Fijian of Others Descent. Since the inclusion criteria included Fiji citizens only, the above 3 groups of people would qualify for the study.
3. Poor education level affecting data has not been discussed in detail as this was not the main objective of this study
4. Gender differences in KAP did not show any significant differences and hence was not discussed.
